# The complete chloroplast genome of *Heterostemma pingtaoi* (Apocynaceae)

**DOI:** 10.1080/23802359.2026.2722710

**Published:** 2026-09-03

**Authors:** Jishi Liu, Yong Yang, Kai Zhang

**Affiliations:** ^a^ Hainan Academy of Forestry (Hainan Academy of Mangroves), Haikou, China; ^b^ Ministry of Education Key Laboratory for Ecology of Tropical Islands, Key Laboratory of Tropical Animal and Plant Ecology of Hainan Province, College of Life Sciences, Hainan Normal University, Haikou, China

**Keywords:** *Heterostemma pingtaoi*, Apocynaceae, chloroplast genome, phylogeny

## Abstract

*Heterostemma pingtaoi*, a species endemic to Hainan Island, China, was discovered and formally described in the family Apocynaceae in 2010. However, genetic and evolutionary information on the genus *Heterostemma* remains poorly understood. Here, we report the complete chloroplast (cp) genome sequence of *H. pingtaoi*. The genome displays the typical quadripartite structure characteristic of dicotyledonous plant cp genomes, with a total length of 162,681 bp and a GC content of 38.25%. The cp genome encodes 131 genes, including 84 protein-coding genes, 8 rRNA genes, and 39 tRNA genes. Phylogenetic analysis based on the complete cp genome sequences confirmed that the genus *Heterostemma* forms a monophyletic group at the base of tribe Ceropegieae (Apocynaceae), with 100% bootstrap support. This study provides essential genomic resources for future taxonomic and evolutionary studies of *Heterostemma* species.

## Introduction

**Figure 1.**
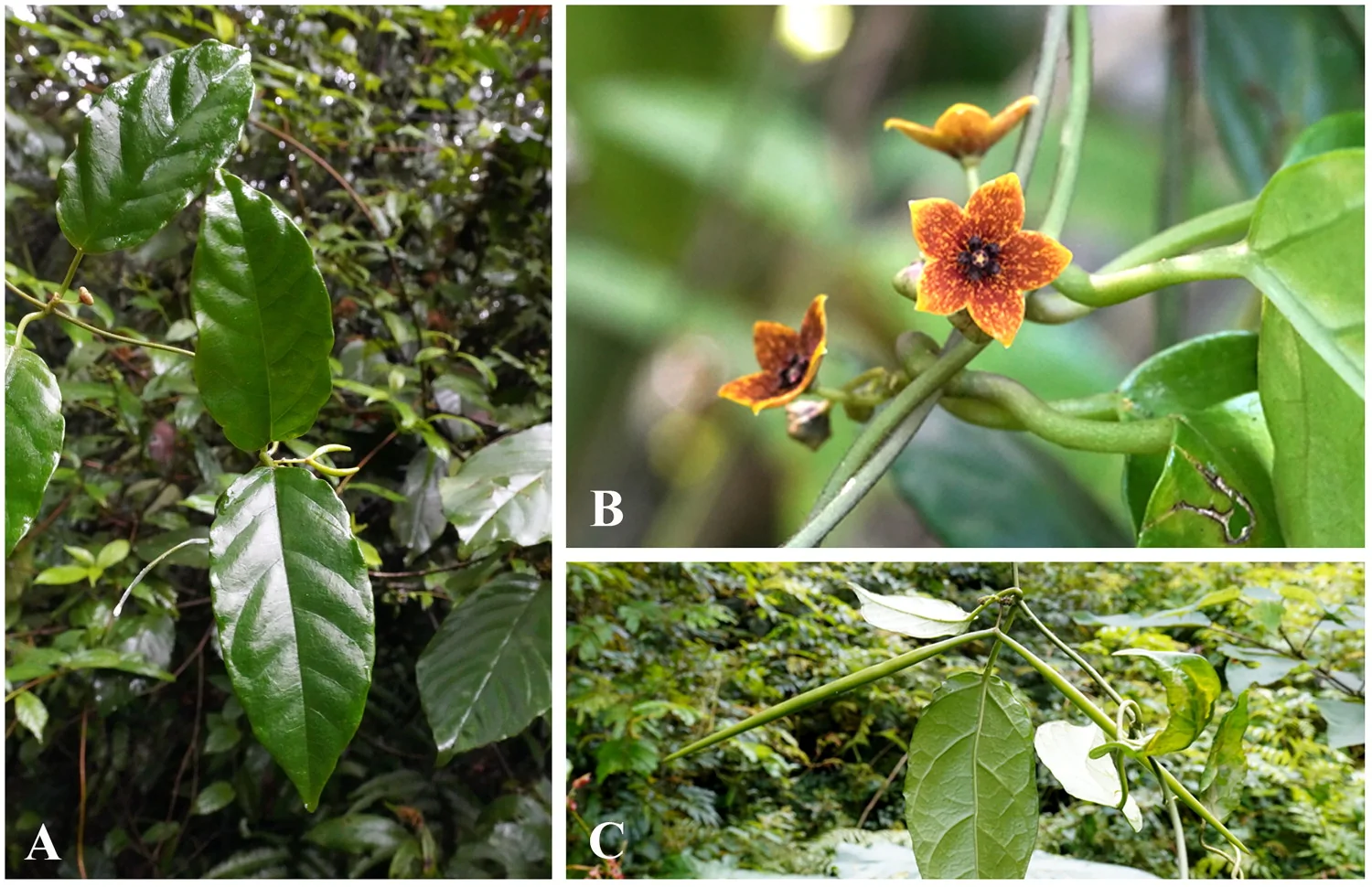
*Heterostemma pingtaoi* in the wild (Jianfengling, Ledong county, Hainan, China). (A) Branch and leaves (adaxial view). (B) Inflorescence. (C) A segment of branch bearing follicles. Photograph by Kai Zhang.

*Heterostemma* Wight & Arnott 1834, a genus of the family Apocynaceae, comprises approximately 30–40 species and is widely distributed across Australia, Bangladesh, China, India, Indonesia, Laos, Malaysia, Myanmar, Nepal, New Guinea, the Philippines, Sri Lanka, Thailand, Vietnam, and the Western Pacific Islands (Swarupanandan et al. 1989; Forster 1992). In China, the genus is currently represented by 12 species, five of which are endemic (Li et al. 1995; Chen and Li 1998; Li et al. 2002; Lin et al. 2010). Among these, *H. pingtaoi* S.Y. He & J.Y. Lin was formally described as a new species in 2010, based on specimens collected from Jianfengling, Hainan Province, China (Lin et al. 2010). Morphologically, *H. pingtaoi* is a woody vine with white latex and can be distinguished by the following diagnostic features: pubescent petioles, elliptical to oblong leaf blades with 3–5 basal veins and 6–9 pairs of lateral veins, and a corolla externally pubescent and yellow-green tinged with mauve; the inner corolla surface is yellow with orange markings, and the wine-red corona lobes are nearly acute at the apex (Figure 1).

To date, knowledge of *H. pingtaoi* has been largely limited to morphological descriptions, while its genomic characteristics and phylogenetic placement remain poorly understood. In this study, we report the first complete chloroplast genome sequence of *H. pingtaoi* and clarify its phylogenetic position among related taxa. Our results provide valuable genomic data and foundational resources for further phylogenetic and evolutionary studies of the genus *Heterostemma*.

## Materials and methods

Fresh leaves of *H. pingtaoi* were collected from a single individual in Jianfengling, Ledong County, Hainan Province, China (N18°44′38.38″, E108°50′39.83″), dried with silica gel, and stored for subsequent DNA extraction. The voucher specimen (Kai Zhang 01480) was deposited at the Hainan Provincial Museum of Biodiversity (Contact: Yukai Chen, chenyukai@hainnu.edu.cn). Total genomic DNA was extracted using a modified CTAB protocol (Doyle and Doyle 1987). Libraries were prepared using the Nextera XT DNA Library Preparation Kit (Illumina, San Diego, CA, USA) according to the manufacturer’s instructions, with an insert size of 350 bp. Libraries that passed quality control were then sequenced on the DNBSEQ-T7 platform (MGI Tech, Shenzhen, China), generating 4.68 Gb of raw sequencing data. Low-quality reads and adapter sequences were removed using fastp v0.23.2 (Chen et al. 2018). The resulting high-quality clean reads were then assembled into the complete chloroplast genome using GetOrganelle v1.7.7.0 (Jin et al. 2020). The Burrow-Wheeler Aligner (BWA) (Li and Durbin 2009) was used to align clean short reads to the assembled chloroplast genome. Per-base coverage depth was calculated with SAMtools (Li et al. 2009), and the coverage plot was generated using a custom Python script. Finally, the chloroplast genome was annotated using PGA (Qu et al. 2019) with *H. oblongifolium* Costantin 1912 (NC_079604.1) as the reference. A circular genome map was generated using OGDRAW v1.3.1 (Greiner et al. 2019), and cis- and trans-splicing genes were analyzed and visualized using CPGView (Liu et al. 2023).

Molecular phylogenetic analysis was conducted using 14 complete chloroplast genomes, including 11 species from seven genera of the tribe Ceropegieae Decne. ex Orb. 1843 as ingroups, and three species in the tribe Marsdenieae Benth. 1868 as outgroups. Except for *H. pingtaoi*, the chloroplast genomes of all other species were downloaded from the NCBI GenBank database. All 14 chloroplast genomes were aligned using MUSCLE v3.8.31 (Edgar 2004) with default parameters and were subsequently manually refined. The maximum-likelihood (ML) tree was reconstructed using IQ-TREE v2.0.3 (Minh et al. 2020) with 1000 bootstrap replicates, and the best-fit evolutionary model (TVM + F + R3) was determined by ModelFinder (Kalyaanamoorthy et al. 2017) based on the Bayesian Information Criterion (BIC).

## Results

**Figure 2.**
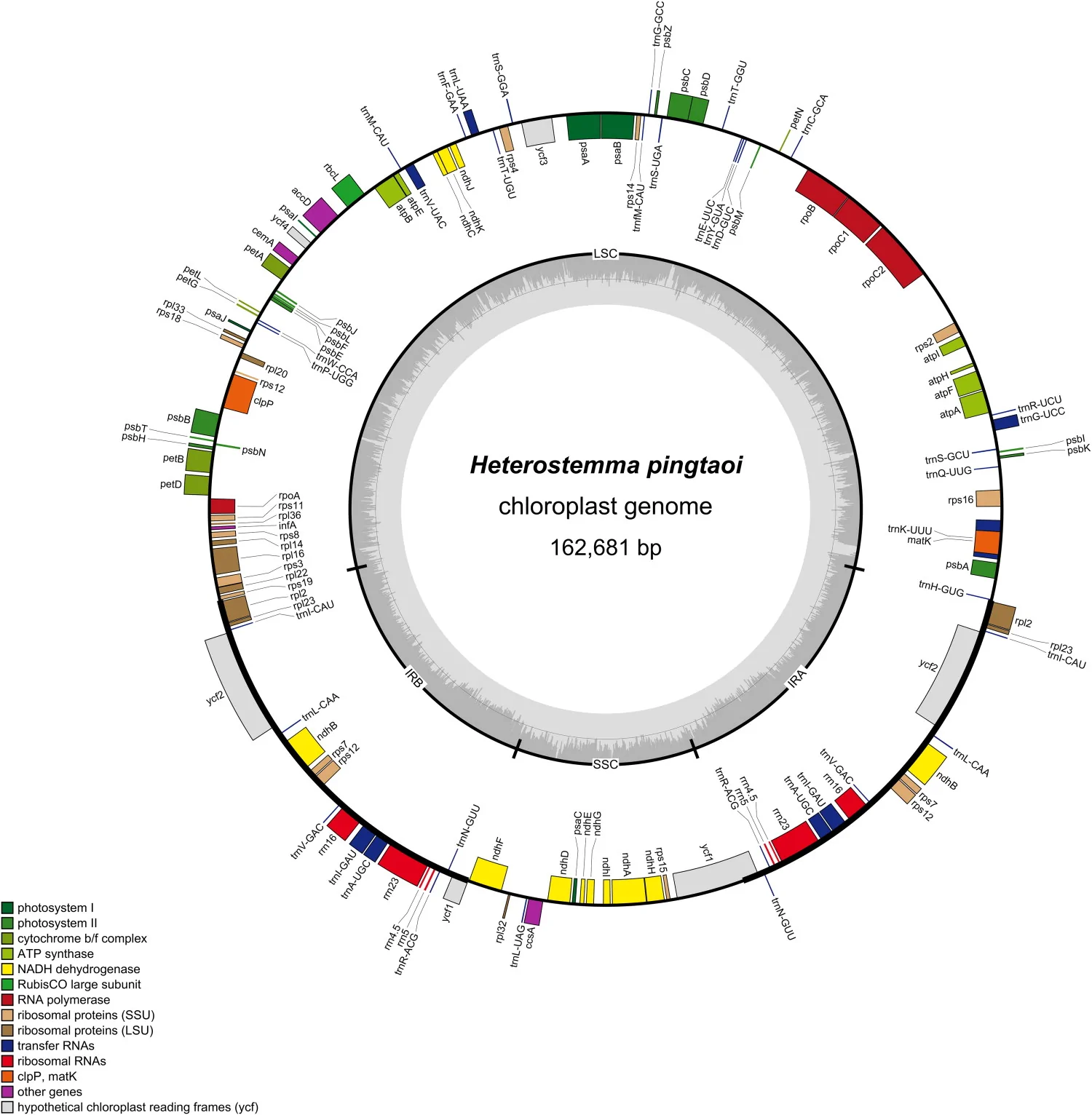
Circular map of the chloroplast genome of *H. pingtaoi*. The inner gray circle represents AT content, while the darker gray circle indicates GC content. Genes transcribed clockwise are shown inside the circle, whereas those transcribed counterclockwise are shown outside. Genes are color-coded according to their functional groups. The inner concentric rings illustrate the typical quadripartite structure of the chloroplast genome, comprising the LSC, SSC, and two IR regions.

The average coverage depth of the assembled chloroplast genome was 2,581.42× (Figure S1), indicating high-quality and relatively even sequencing coverage across the genome. Structural analysis revealed that the complete chloroplast genome of *H. pingtaoi* is a typical quadripartite circular molecule of 162,681 bp (Figure 2), with an overall GC content of 38.25%. It comprises a large single-copy (LSC) region of 93,379 bp, a small single-copy (SSC) region of 18,350 bp, and a pair of inverted repeat (IR) regions of 25,476 bp each. Genome annotation identified a total of 131 genes, including 84 protein-coding genes (PCGs), 8 ribosomal RNA (rRNA) genes, and 39 transfer RNA (tRNA) genes. We identified 11 unique cis-splicing genes in the chloroplast genome (Figure S2). Among these, nine genes (*rps16*, *atpF*, *rpoC1*, *petB*, *petD*, *rpl16*, *rpl2*, *ndhB*, and *ndhA*) each contained a single intron, while *clpP* and *ycf3* each harbored two introns. The *rpl2* and *ndhB* genes each had two identical copies located in the inverted repeat regions, accounting for the 13 cis-splicing gene structures shown in Figure S2. In addition, the *rps12* gene was identified as the only trans-splicing gene with three exons (Figure S3). Phylogenetic analysis showed that the two *Heterostemma* species formed a highly supported monophyletic group and were located at the base of the tribe Ceropegieae. The backbone of Ceropegieae, represented by 11 species from three subtribes, was well resolved with strong support.

## Discussion and conclusion

Meve and Liede (2004) divided the tribe Ceropegieae into four subtribes, namely Anisotominae Meve & Liede 2004, Heterostemminae Meve & Liede 2004, Leptadeniinae Meve & Liede 2004, and Stapeliinae G. Don 1838, based on non-coding chloroplast DNA markers (*trnT-L* and *trnL-F* spacers, and the *trnL* intron). Although representatives of subtribe Anisotominae were not included in our sampling, the phylogenetic tree based on complete chloroplast genomes further confirmed that Heterostemminae, Leptadeniinae, and Stapeliinae were each monophyletic. The genus *Heterostemma* was resolved as sister to all other genera in the tribe Ceropegieae, which is consistent with the findings of Wang et al. (2023) and Yao et al. (2025).

**Figure 3.**
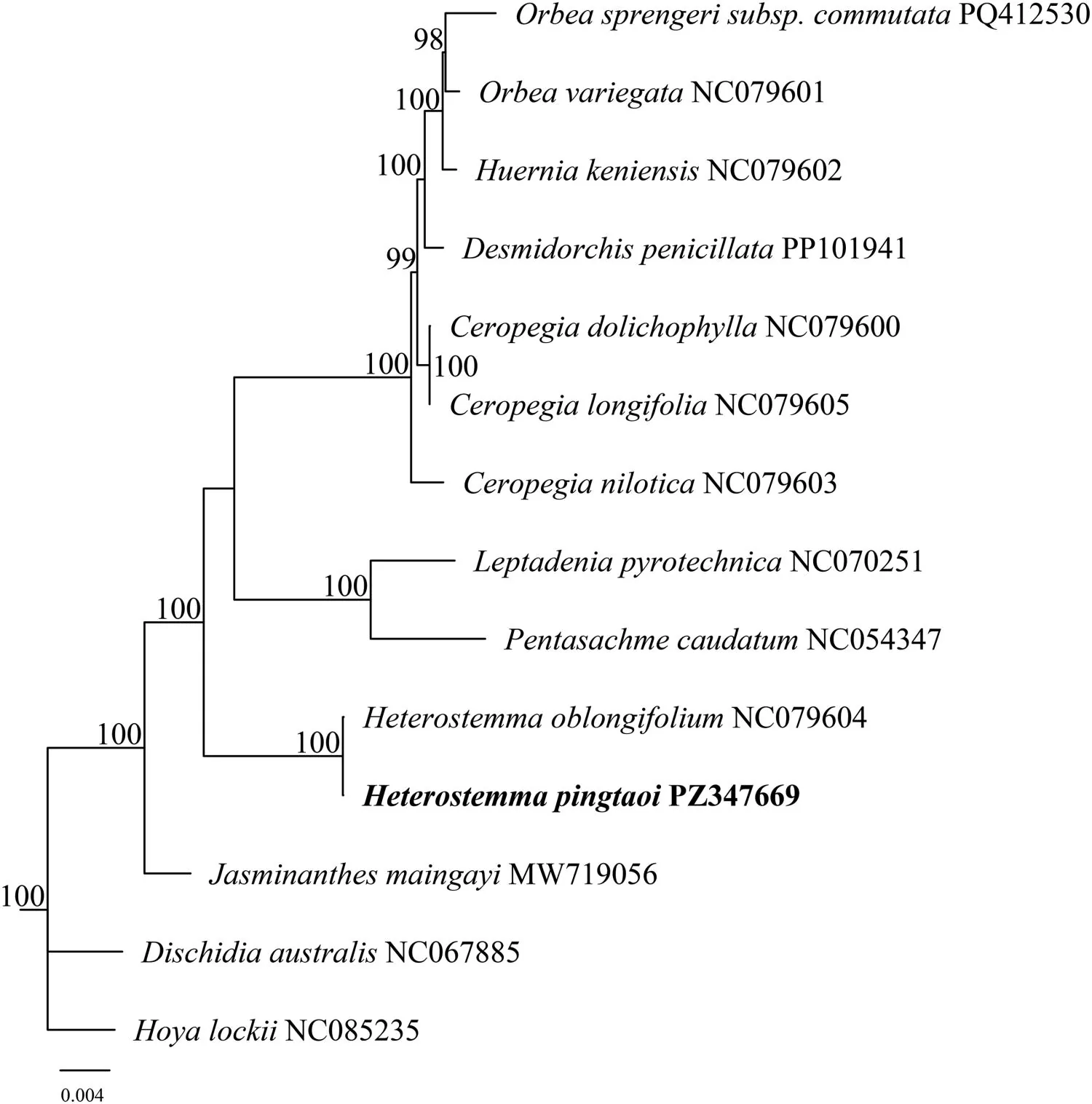
The maximum-likelihood phylogenetic tree based on the complete genome sequences of *H. pingtaoi* and 13 other species. Bootstrap values are shown at the nodes, and the Latin names and GenBank accession numbers of the species are shown at the end of each branch. The 13 chloroplast genome sequences were retrieved from GenBank under the following accession numbers: *H. oblongifolium* NC_079604 (Wang et al. 2023), *Pentasachme caudatum* NC_054347 (Miao et al. 2021), *Leptadenia pyrotechnica* NC_070251, *Ceropegia dolichophylla* NC079600, *C. longifolia* NC_079605, *C. nilotica* NC_079603, *Desmidorchis penicillata* PP101941 (Alharbi and Albokhari 2025), *Huernia keniensis* NC_079602, *Orbea variegata* NC_079601, *O. sprengeri* subsp. *commutata* PQ412530 (Alharbi 2026), *Hoya lockii* NC_085235 (Nguyen et al. 2026), *Dischidia australis* NC_067885, *Jasminanthes maingayi* MW719056 (Rodda and Niissalo 2021).

As shown in Figure 3, *H. pingtaoi* and *H. oblongifolium* clustered together with 100% bootstrap support, and their two terminal branches were extremely short, indicating a very close phylogenetic relationship between these two species. Chloroplast genome data alone could not effectively distinguish these two taxa. Consistently, no distinct morphological differences were observed between the two species during our field investigations; therefore, further studies are required to clarify their taxonomic status.

In this study, we successfully assembled and annotated the complete chloroplast genome of *H. pingtaoi* for the first time. The results revealed its close phylogenetic relationship with *H. oblongifolium*, and confirmed the evolutionary relationships among subtribes Heterostemminae, Leptadeniinae, and Stapeliinae. This study not only enriched the genomic resources available for *Heterostemma* but also provided a molecular foundation for future taxonomic revision, the development of conservation strategies, and adaptive evolution studies.

## Supplementary Material

Supplemental Material

Supplemental Material

Supplemental Material

## Data Availability

The complete chloroplast genome sequence of *H. pingtaoi* is deposited in NCBI GenBank under accession number PZ347669. The associated BioProject, Bio-Sample, and SRA numbers are PRJNA1459318, SAMN57545095, and SRR38320946, respectively.
