# Supplementary material for: The complete chloroplast genome of Heterostemma pingtaoi (Apocynaceae)
Source: Mitochondrial DNA B Resour. 2026 Sep 3;11(10):1112–6. doi: 10.1080/23802359.2026.2722710 (PMC13543828; doi:10.1080/23802359.2026.2722710)
Supplement: Supplementary file 1 [file TMDN_A_2722710_SM6519.pdf]

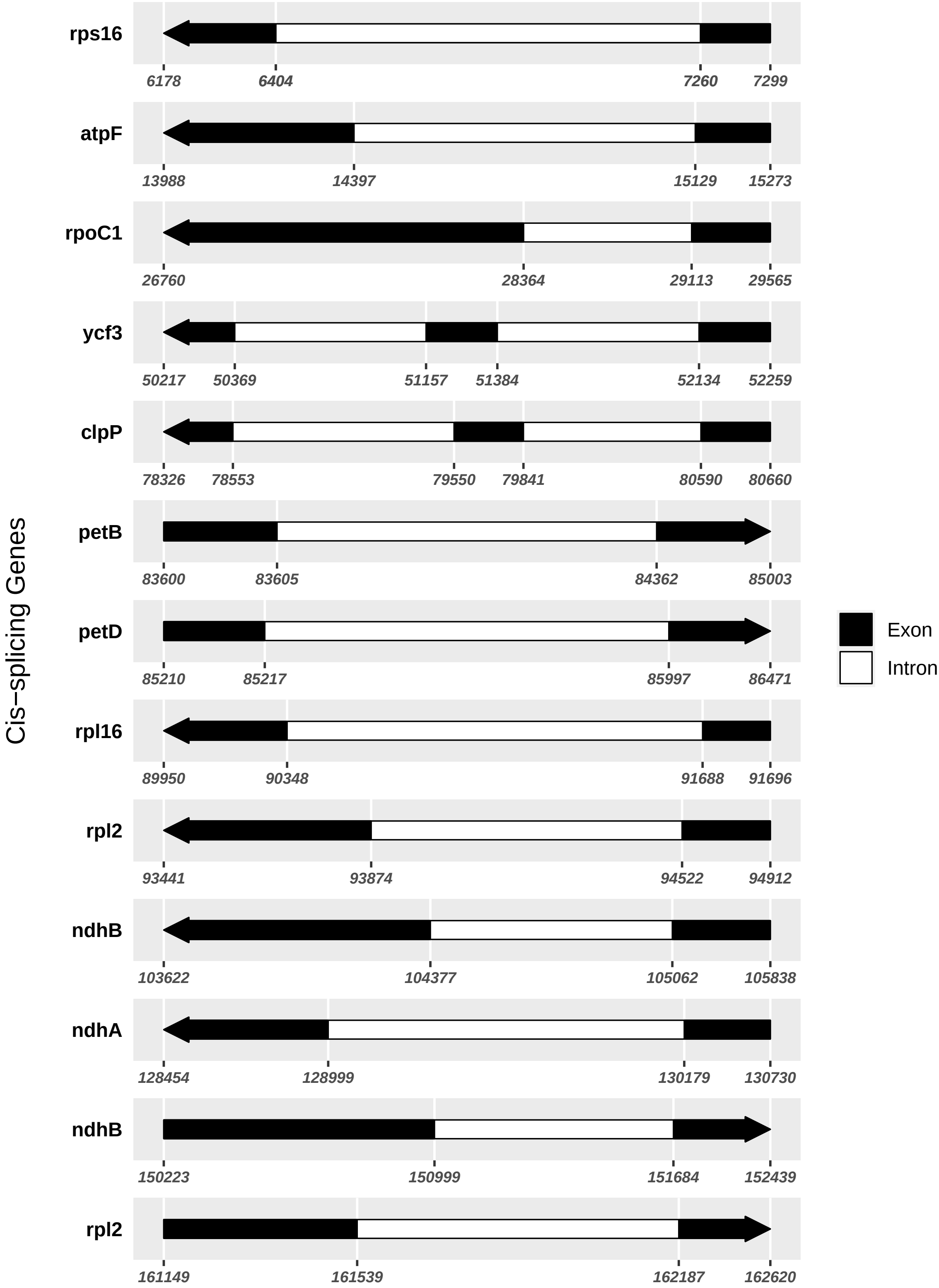

Cis−splicing Genes
rps16
6178
6404
7260
7299
atpF
13988
14397
15129
15273
rpoC1
26760
28364
29113
29565
ycf3
50217
50369
51157
51384
52134
52259
clpP
78326
78553
79550
79841
80590
80660
petB
83600
83605
84362
85003
petD
85210
85217
85997
86471
rpl16
89950
90348
91688
91696
rpl2
93441
93874
94522
94912
ndhB
103622
104377
105062
105838
ndhA
128454
128999
130179
130730
ndhB
150223
150999
151684
152439
rpl2
161149
161539
162187
162620
Exon
Intron
