# Supplementary material for: The complete chloroplast genome of Heterostemma pingtaoi (Apocynaceae)
Source: Mitochondrial DNA B Resour. 2026 Sep 3;11(10):1112–6. doi: 10.1080/23802359.2026.2722710 (PMC13543828; doi:10.1080/23802359.2026.2722710)
Supplement: Supplementary file 2 [file TMDN_A_2722710_SM6523.pdf]

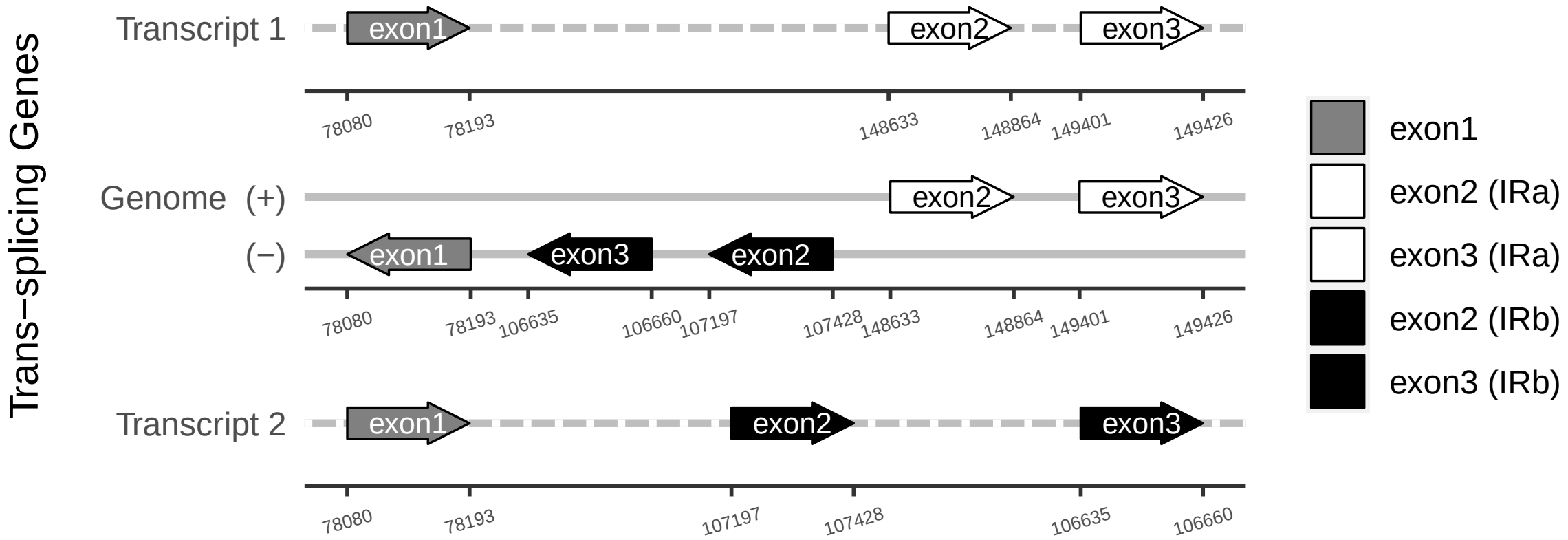
Trans−splicing Genes
Transcript 1
exon1
exon2
exon3
78080
78193
148633
148864
149401
149426
Genome (+)
exon2
exon3
(−)
exon1
exon3
exon2
78080
78193
106635
106660
107197
107428
148633
148864
149401
149426
Transcript 2
exon1
exon2
exon3
78080
78193
107197
107428
106635
106660
exon1
exon2 (IRa)
exon3 (IRa)
exon2 (IRb)
exon3 (IRb)
